# Utility of the Parkland Grading Scale to determine intraoperative challenges during laparoscopic cholecystectomy: a validation study on 206 patients at an academic medical center in Nepal

**DOI:** 10.1186/s13037-023-00364-x

**Published:** 2023-05-24

**Authors:** Anup Shrestha, Abhishek Bhattarai, Kishor Kumar Tamrakar, Manoj Chand, Samjhana Yonjan Tamang, Sampada Adhikari, Harish Chandra Neupane

**Affiliations:** 1 Department of Surgery, Chitwan Medical College and Teaching Hospital, Chitwan, Nepal; 2 Department of Surgery, Indira Gandhi Memorial Hospital, Male, Maldives; 3 School of Medicine, Chitwan Medical College and Teaching Hospital, Chitwan, Nepal

**Keywords:** Difficulty, Laparoscopic cholecystectomy, Parkland Grading Scale, Utilization

## Abstract

**Background:**

Most of the scoring systems to predict difficult laparoscopic cholecystectomy are based on pre-operative clinical and radiological findings. Recently the Parkland Grading Scale system was introduced as a simple intra-operative grading scale. This study aims to utilize the Parkland Grading Scale system to assess the intraoperative challenges during laparoscopic cholecystectomy.

**Method:**

This was a prospective, cross-sectional study done at Chitwan Medical College and Teaching Hospital, Chitwan, Nepal. All the patients underwent laparoscopic cholecystectomy from April 2020 to March 2021. Based on the initial intra-operative finding, Parkland Grading Scale was noted and at the end of the surgery, the level of difficulty was given by the operating surgeon. All the pre-operative, intra-operative, and post-operative findings were compared with the scale.

**Results:**

Out of 206 patients, there were 176 (85.4%) females, and 30 (14.6%) males. The median age was 41 years (Range 19–75). The median body mass index was 23.67 kg/m2. There were 35(17%) patients with a history of previous surgery. The rate of conversion to open surgery was 5.8%. According to Parkland Grading Scale, 67(32.5%), 75(36.4%), 42(20.4%), 15(7.3%), and 7(3.4%) were graded as grade 1, 2, 3, 4, and 5 respectively. There was a difference in the Parkland grading scale in patients with a history of acute cholecystitis, gallbladder wall thickness, pericholecystic collection, stone size, and body mass index (p < 0.05). The total operative time, level of difficulty in surgery, rate of help needed from colleagues or replacement as the main surgeon, bile spillage, drain placement, gallbladder decompression, and conversion rate all increased with an increase in scale (p < 0.05). There was a significant increase in the development of post-operative fever, and post-operative hospital stay as the scale increased (p < 0.05). The Tukey-Kramer test for all pair-wise comparisons revealed that each grade was significantly different from each other (p < 0.05) on the difficulty of surgery except for grade 4 from 5.

**Conclusion:**

Parkland Grading Scale system is a reliable intra-operative grading system to assess the difficulty in laparoscopic cholecystectomy and helps the surgeon to change the strategy of surgery. An increase in scale is associated with an increased difficulty level of the surgery.

## Background

Gallstones disease (GSD) is one of the most common biliary pathology [1]. It is one of the common reasons for hospital admission in the department of surgery [2]. The overall prevalence of gallstones worldwide is 10–20% [3]. The prevalence of GSD in Nepal is 4.87% [4]. Surgery is the mainstay for the management of symptomatic GSD [5]. Laparoscopic cholecystectomy (LC) have been established as a gold standard for the management of symptomatic GSDs [6].There are various advantages of LC like it decreases postoperative pain, decreases the need for postoperative analgesia, shortens the hospital stay from 1 week to less than 24 h, and returns the patient to full activity within 1 week [7–9]. The adoption of universal culture of safety in LC should always be maintained [10]. Bile duct injury (BDI) is a catastrophic complication associated with significant peri-operative morbidity and mortality, reduced long-term survival and quality of life, and high rates of subsequent litigation [11, 12].

Every LC is not always easy to perform [13]. There are various factors for the assessment of difficulty in LC like difficult access, difficult grasping and retraction of the gallbladder (GB), difficult dissection of Calot’s triangle, abnormal anatomy, difficult retrieval of the specimen, and total operative time > 180 min [14–16]. Various scoring system has been developed to predict difficult LC [17–20]. Most of the scoring systems are based on pre-operative clinical and radiological findings while some also included intra-operative findings [17–20]. These scoring systems are complex and includes multiple factors which may not be feasible in practical [18, 19, 21, 22]. One of the major factors to predict difficulty in LC is inflammation of the GB [18]. Severity of gallbladder inflammation cannot be made out clearly until gall bladder is visualized during surgery [10]. In 2018, Parkland Grading Scale system was introduced by Madni et al. [23]. This is a simple intra-operative based grading scale system based on anatomy and inflammatory changes of gallbladder which is seen on initial intra-operative view during LC [23]. Very few studies have been done based on grading scale [24, 25]. Till now only one study has been done from Nepal which is based on intra-operative grading scale [26]. This study aims to utilize the Parkland Grading Scale system to assess the intraoperative challenges during LC.

## Methods

This was a descriptive cross-sectional study done at department of General Surgery, Chitwan Medical College Teaching Hospital (CMCTH), Bharatpur, Nepal. The Institutional Review Committee of CMCTH approved this prospective observational study (Reference No: CMC-IRC/077/078–228), Date:22/03/2020). Written consent was given by patients for the information to be used for the research. It was done from March 2020 to February 2021. All the patients presented to the surgical out-patient department of CMCTH with the clinical diagnosis of symptomatic cholelithiasis and its associated complications like acute cholecystitis, resolved biliary pancreatitis and chronic cholecystitis. Complete enumeration technique was used to collect data. All the cases fulfilling the inclusion criteria were included in the study.

### Inclusion criteria

- All patient undergoing LC for symptomatic cholelithiasis, acute cholecystitis, resolved biliary pancreatitis and chronic cholecystitis.

### Exclusion criteria

- Age < 18.
- Lap to open conversion due to equipment failure.
- Choledocholithiasis.
- Presence GB polyps.
- Concomitant another procedure.
- Patients not willing to be part of the study.

All those patients presenting with clinical features of GSD in CMCTH surgical out-patient department were enrolled in this study using the above-mentioned inclusion and exclusion criteria. Clinical assessment of the case (full history and physical examination) was done by the residents and the consultant on duty. GSD was confirmed with ultrasonographic features of highly reflective echogenic focus within gallbladder lumen, normally with prominent posterior acoustic shadowing regardless of pathological type and gravity-dependent movement often seen with a change of patient position.

The clinical parameters like age, sex, body mass index (BMI), history of previous abdominal surgery, history of previous hospitalization for acute cholecystitis and pancreatitis, history of previous abdominal surgery or endoscopic retrograde cholangio-prancreaticography (ERCP), palpable GB, and ultrasound findings such as gall bladder wall thickness, CBD diameter, condition of liver (fatty or normal) sludge and pericholecystic collections was noted down in proforma. Laboratory investigation like white blood cell (WBC) count, hemoglobin (Hb%), PT (prothrombin time), total bilirubin, direct bilirubin, aspartate transaminase (AST), alanine transaminase (ALT), and alkaline phosphatase (ALP) were noted.

### Grading of Parkland Grading Scale

The operative procedure was done under standard hospital protocol for LC. The main surgeon graded the Parkland Grading Scale on the basis of initial view of the gallbladder. The initial view was defined as follows:

- If the GB was visualized easily, it was grasped and retracted cephalad giving the “initial view”.
- If severe inflammation was present which limited mobilization or the ability to visualize the GB, the “initial view” was defined as the view of the inflamed area.

The grading was done as follows according to Parkland Grading Scale system [23]:

Grade 1: normal GB/no adhesions

Grade 2: minor adhesions at the neck

Grade 3: presence of any of the following: hyperemia, peri-cholecystic fluid, adhesions to the body, distended GB

Grade 4: presence of any of the following: Adhesions obscuring majority of GB or Grade I–III with abnormal liver anatomy, intrahepatic GB, or impacted stone (Mirizzi).

Grade 5: presence of any of the following: Perforation, necrosis, inability to visualize the GB due to adhesions.

At the end of the surgery, the difficulty level was recorded as level 1, 2, 3, 4, and 5 for very easy, easy, normal, difficult, and very difficult respectively by the main operating surgeon. Intra-operative parameters like Parkland Grading Scale, Difficulty level of surgery, BDI, artery injury, right hepatic artery anomaly, Bile spillage, pericholecystic adhesions, manual GB decompression, Thick-wall of GB > 4 mm, intra-operative bleeding, drain placement, difficult to extract GB from port, help needed from colleagues, conversion to open surgery and total operative time were recorded. The total operative time was calculated from the time of skin incision to the end of skin closure. The difficulty level of surgery was decided by the operating surgeon at the end of the surgery according to 5-point Likert Scale [27]. Post-operatively total hospital stay, post-operative bile leak, surgical site infection (SSI), fever and pneumonia were recorded. Bile leak was defined using a standardized definition from the International study Group of Liver Surgery [28].

### Statistical analysis

All the statistical analysis was done by using Statistical Package for Social Sciences (SPSS) version 16. The Fisher’s Exact Test was used to compare the categorical variables while the one-way ANOVA or (Kruskal –Wallis test) was used to compare continuous or ordinal variables. The Fisher’s Exact test was used to test association between Parkland Grading Scale and categorical variable because more than 20% of cells had expected frequencies < 5. Kruskal Wallis test was used to test association between Parkland Grading Scale and continuous and ordinal variable because it is used to compare three or more groups on a dependent variable that is measured on at least an ordinal level. Jonckheere-Terpstra test was used to test association between Parkland Grading Scale and difficulty level of the surgery. The Tukey-Kramer test was used for pairwise comparisons between each grade. P-value < 0.05 was considered statistically significant.

## Results

A total of 206 patients were graded utilizing Parkland Grading Scale system. The patient’s demographic, peri-operative and post-operative characteristics are illustrated in Table 1. The median BMI was 23.67 kg/m^2^ (range 18.3–36.1 kg/m^2^). Pericholecystic collection was seen in 5 patients (2.4%). Thick GB wall > 4 mm was noted in 180(87.4%) patients. Twelve (5.8%) patients underwent laparoscopic converted to open surgery. There were 2 cases of duct injury, one sectoral duct injury and the other complete transection of the CBD. The patient underwent hepticojejunostomy for complete bile duct transaction. Intra-operative bleeding and bile spillage was seen in 14(6.8%) and 44(21.4%) respectively. Drain was placed in 19.4%. In 20 cases the surgery was overtaken by the senior surgeon. The median total operative time was 55 min. 82(39.8%), 59(28.6%), 31(15%), 23(11.2%), and 11(5.3%) of surgery was labeled as very easy, easy, normal, difficult and very difficult according to Likert scale by the main operating surgeon. The median total post-operative stay was 2 days. 3 patients had post-operative bile leak and all the patients were successfully treated by conservative management. Similarly, five patients had SSI which were all superficial and treated in out-patient basis. 5 patients developed post-operative pneumonia and among them one patient was admitted in surgical intensive care unit for shortness of breath. Similarly, 11 patients had post-operative fever. There was no in-hospital mortality recorded.

**Table 1:** Patient demographic, peri-operative, and post-operative characteristics

Gender	
Male Female	30(14.6) 176(85.4)
Median Age, years (range)	41(19–75)
Parkland Grading Scale Grade	
Grade 1 Grade 2 Grade 3 Grade 4 Grade 5	67(32.5) 75(36.4) 42(20.4) 15(7.3) 7(3.4)
Diagnosis	
Symptomatic cholelithiasis Acute Cholecystitis Chronic Cholecystitis Resolved Biliary Pancreatitis	169(82) 13(6.3) 15(7.3) 9(4.4)
History of Cholecystitis	20(9.7)
History of ERCP	1(0.5)
Previous abdominal surgery Upper abdomen surgery Lower abdomen surgery	35(17) 4(1.9) 31(15.1)
BMI category	
Under weight Normal weight Overweight Obese	3(1.5) 122(59.2) 75(35.4) 8(3.9)
Intra-abdominal adhesion	
Yes No	78(37.9) 128(62.1)
Manual GB decompression	
Yes No	15(7.3) 191(92.7)
Partial Cholecystectomy	3(1.4)
Converted to open surgery	12(5.8)
Help needed from colleagues	20(9.7)
Operative time, median(range) minutes	55(35–360)
Difficulty level of surgery	
Level 1-Very easy Level 2- Easy Level 3- Normal Level 4-Difficult Level 5-Very Difficult	82(39.8) 59(28.6) 31(15) 23(11.2) 11(5.3)

There was no significant difference in pre-operative parameters like age, WBC count total bilirubin, ALT, AST, ALP, and history of previous surgery in Parkland Grading Scale. There was difference in Parkland Grading Scale in thickness of GB wall, incidence of pericholecystic collection, history of acute cholecystitis and BMI (p < 0.05) (Table 2). Regarding intra-operative events and finding, the stone size, total operative time, level of difficulty in surgery, help by colleagues or replacement as main surgeon, GB decompression, bile spillage and conversion rate all increased with increase in Parkland Grading Scale. There was no significant difference in intra-operative bleeding and bile duct injury (Table 3). There was significant increase in development of post-operative fever, total operative time and post-operative hospital stay as the Parkland Grading Scale increases (Table 4).

**Table 2:** Association of Parkland Grading Scale with Pre-Operative Parameters n = 206

	Parkland Grading Scale					p value
	1 (n = 67)	2(n = 75)	3(n = 42)	4(n = 15)	5(n = 6)	
Age, median	40 (21–68)	46 (15–67)	43 (23–67)	46 (30–75)	56 (37–75)	0.296^*^
WBC, median (range)	7000 (4200–10800)	6500 (4400–11300)	6650 (4300–13000)	6400 (4500–8800)	7400 (4500–8800)	0.289^*^
Total Bilirubin, median(range)	0.6 (0.3–1.7)	0.6 (0.36-5)	0.6 (0.4–2.5)	0.5 (0.4–1.8)	0.6 (0.4–0.8)	0.420^*^
ALT	34 (17–95)	34 (16–143)	35 (20–132)	29 (13–138)	37 (22–77)	0.443^*^
AST	28 (12–67)	31 (12–67)	28.5 (17–123)	34 (14–124)	41 (22–58)	0.809^*^
BMI, median(range)	23.2 (18.3–29.4)	23.4 (18.4–35.2)	25.1 (20.1–35.2)	25.8 (21.1–36.1)	26.9 (21.3–27.9)	0.006^*^
Largest stone, mm(range)	10.5 (4–26)	12 (2–30)	10.2 (5–37)	15 (3–40)	10 (12–22)	0.005^*^
H/o Cholecystitis, n(%)						< 0.001^**^
Yes No	2(3) 65(97)	2(2.7) 73(97.3)	11(26.2) 31(73.8)	3(20) 12(80)	2(28.6) 5(71.4)	
h/o of previous abdominal surgery, n(%)						0.350^**^
Yes No	15(22.4) 52(77.6)	10(13.3) 65(86.7)	6(14.3) 36(85.7)	4(26.7) 11(73.3)	- 7(100)	
GB thickness, n(%)						< 0.001^**^
< 4mm > 4mm	65(97.1) 2(2.9)	74(98.6) 1(2.4)	30(71.4) 12(28.6)	8(53.3) 7(46.7)	1(14.3) 6(85.7)	
Peri-cholecystic collection, n(%)						0.003^**^
Yes No	- 67(100)	1(1.3) 74(98.7)	1(2.4) 41(97.6)	1(6.7) 14(93.3)	2(28.6) 5(71.4)	

^*^Kruskal –Wallis test was used to assess the association of grade with continuous measures

^**^Fisher’s Exact Test was used to test the association of grade with binary (categorical) variables

**Table 3:** Association of Parkland Grading Scale with Intra-Operative Events n = 206

	Parkland Grading Scale					p value
	1 (n = 67)	2(n = 75)	3(n = 42)	4(n = 15)	5(n = 6)	
GB decompression,						< 0.001^*^
Yes No	- 67(100)	1(1.3) 74(98.7)	10(23.8) 32(76.2)	3(20) 12(80)	1(14.3) 6(85.7)	
Bile spillage						< 0.001^*^
Yes No	5(7.5) 62(92.5)	15(20) 60(80)	11(26.7) 31(73.8)	10(66.7) 5(33.3)	3(42.9) 4(57.1)	
Intra op bleeding						0.245^*^
Yes No	2(3) 65(97)	5(6.7) 70(96.3)	4(9.5) 38(90.5)	2(13.3) 13(86.7)	1(14.3) 6(85.7)	
Help from seniors						< 0.001^*^
Needed Not needed	2(3) 65(97)	5(6.7) 70(93.3)	1(2.4) 41(97.6)	7(46.7) 8(53.3)	5(71.4) 2(28.6)	
Bile duct injury						0.243^*^
Yes No	0 67(100)	1(1.3) 74(98.7)	0 42(100)	1(6.7) 14(93.30	0 7(100)	
Drain placed						< 0.001^*^
Yes No	1(1.5) 66(98.50	6(8) 69(92)	15(35.7) 27(64.3)	11(73.3) 4(26.7)	7(100) 0	
Converted to open surgery, n(%)						< 0.001^*^
Yes No	1(1.5) 66(98.5)	1(1.3) 74(98.7)	3(7.1) 39(92.9)	4(26.7) 11(73.3)	3(42.9) 4(57.1)	
OT Time, median (range) minutes	50(35–180)	55(35–180)	75(35–360)	120(50–240)	150(120–240)	< 0.001^**^
Difficulty level of surgery						< 0.001^***^
Very easy Easy Normal Difficult Very difficult	48(71.6) 18(26.9) - 1(1.5) -	33(44) 27(36) 12(16) 2(2.7) 1(1.3)	1(2.4) 12(28.6) 16(38.1) 11(26.2) 2(4.8)	- 2(13.3) 3(20) 5(33.3) 5(33.3)	- - - 4(57.1) 3(42.9)	

^*^Fisher’s Exact Test was used to test the association of grade with binary (categorical) variables

^**^Kruskal –Wallis test was used to assess the association of grade with continuous measures

^***^ Jonckheere-Terpstra test was used for doubly-ordered categorical data

**Table 4:** Association of Parkland Grading Scale and Post-Operative Outcome n = 206

	Parkland Grading Scale					p value
	1	2	3	4	5	
SSI, n(%),						< 0.001^*^
Yes No	- 67(100)	- 75(100)	2(4.7) 40(95.3)	2(4.8) 13(95.2)	1(14.3) 6(85.7)	
Post-op. pneumonia,n(%)						0.139^*^
Yes No	- 67(100)	3(4) 75(96)	1(2.4) 40(97.6)	- 13(100)	1(14.3) 6(85.7)	
Post-op fever, n(%)						0.003^*^
Yes No	2(3) 1(97)	1(1.3) 75(98.3)	3(7.1) 39(92.9)	4(26.7) 11(73.3)	1(14.3) 6(85.7)	
Post-operative bile leak						0.244^*^
Yes No	- 67(100)	1(1.3) 74(98.7)	1(7.1) 41(92.9)	1(26.7) 14(73.3)	0 7(100)	
Length of post-operative stay, median (range)	2(1–5)	2(2–18)	2(2–8)	4(2–9)	4(2–5)	< 0.001^**^

^*^ Fisher’s Exact Test was used to test the association of grade with binary (categorical) variables

^**^Kruskal –Wallis test was used to assess the association of grade with continuous measures

The Tukey-Kramer test for all pairwise comparisons revealed that each Grade [1–5] was significantly different from each other (at p < 0.05) on difficulty of surgery except for grade 4 from 5. Regarding length of surgery there was no statistical significance between 2 groups; grade from grade 2 and grade 4 from grade 5 while rest of there was significant difference between rests of the grades. In total post-operative hospital stay, grade 1 was significant different from grade 4 and 5; grade 2 from grade 4, grade 3 from 4; grade 4 from grade 1, 2, 3 and grade 5 from grade 1.

## Discussion

The main aim of this study was to assess the utilization of Parkland Grading Scale system to determine the difficulty level during LC. Our study included 206 patients who underwent LC. Only in 1.5% of the Parkland Grading Scale grade 1 patient, the difficulty of surgery was rated as difficult. Among Parkland Grading Scale grade 2, 2.7% were rated as difficult and 1.3% were rated as very difficult respectively. In Parkland Grading Scale grade 3, 26.2% and 4.85% of the surgeries were rated as difficult and very difficult respectively. In Parkland Grading Scale grade 4, the difficulties of surgery were rated as difficult and very difficult in 33.3% each. In Parkland Grading Scale grade 5, 57.1% was labeled as difficult while 42.9% was labeled as very difficult. The results showed that the rate of surgical difficulty level increases with the increase in Parkland Grading Scale grade (p value < 0.001). Till now only 4 studies has been published in the literature [26, 29–31]. Only Madni et al. have categorized the difficulty of surgery for each Parkland Grading Scale grade [31]. They reported in Parkland Grading Scale grade 1, only 1.7% was rated as difficult. In Parkland Grading Scale grade 2, 4.4% were rated as difficult. In Parkland Grading Scale grade 3, 12.7% and 3.9% were rated as difficult and very difficult respectively. In Parkland Grading Scale grade 4, 50% were rated as difficult while 14.3% were rated as very difficult. In Parkland Grading Scale grade 5, 32.4% were rated as difficult and 59.5% were rated as very difficult. There was significant statistical difference in the difficulty of surgery as Parkland Grading Scale grade increases which were consistent with the current study. Above findings suggest that Parkland Grading Scale system is feasible to assess the difficulty level of LC. If the resident or junior surgeon is performing the LC, it guides them to seek early help from the senior for GB with high Parkland Grading Scale grades. It also guides the senior experienced surgeon for early conversion into open surgery and early replacement as main operating surgeon for GB with the high Parkland Grading Scale grades.

In our study, there was significant association of Parkland Grading Scale with the GB decompression, bile spillage, help needed from senior, drain placement, conversion rate and operative time. Our overall conversion rate was 5.8% and only 3(1.4%) patients underwent subtotal cholecystectomy. Madni et al. reported only 9(2.8%) were converted to open surgery which was lower than our report [31]. The possible reason may be due to differences in the expertise of a surgeon. In our present series, the conversion rate in Parkland Grading Scale grade 1, 2, 3, 4, and 5 was 1.5%, 1.3%, 7.1%, 26.7%, and 42.9% respectively. This reports that as the Parkland Grading Scale grade increases the conversion rate also increases. This suggests the increase in level of difficulty of surgery as the Parkland Grading Scale grade increases. Madni et al. reported that, there was no conversion to open surgery in Parkland Grading Scale grade 1, 2, and 3 while the open conversion rate in Parkland Grading Scale grade 4 and 5 was 3.8% and 21.6% respectively [31]. Abdul et al. reported no cases were converted to open surgery [30]. Baral et al. also reported there was no open conversion in Parkland Grading Scale grade 1 and 2 while the open conversion rate for Parkland Grading Scale grade 3, 4, and 5 were 7.6%, 25%, and 100% respectively [26]. Even the open conversion rate for different Parkland Grading Scale grade was variable, there was significant statistical difference in open conversion rate as the Parkland Grading Scale grade increases which was consistent with our study. (p < 0.001) (Table 3). This report suggests that during LC when high Parkland Grading Scale grade is graded we have to alert the whole operating team for possible conversion into open surgery. This also predicts increase in the difficulty level of the surgery. The present study reports that there was significance difference of total operative time with the increase in Parkland Grading Scale grade. The median time for Parkland Grading Scale grade 1, 2, 3, 4, and 5 was 50 min, 55 min, 75 min, 120 and 150 min respectively. The time taken for higher grade is more than 90 min which is considered to be a risk factor for morbidity [32].Similarly Madni et al., Abdul et al. and Baral also reported statistically significant difference in total operative time in relation to different Parkland Grading Scale grade [26, 30, 31]. This finding suggests that as the Parkland Grading Scale grade increases the total operative time increases which will subsequently increase the difficulty level of the surgery.

Several studies have reported various pre-operative scores to predict difficult LC. In their studies they have found that age, male gender, WBC count, BMI, GB inflammation, GB wall thickness, history of abdominal surgery and previous admission for cholecystitis as the pre-operative factors to predict difficulty LC [18, 19, 21, 33–36]. Among them Strasberg et al. has pointed out GB inflammation as the most common reason for conversion to open cholecystectomy [34].In our present research, male, BMI, history of cholecystitis, GB wall thickness and pericholecystic collection were significantly associated with higher grade of Parkland Grading Scale whereas age, WBC count, total bilirubin, ALT, AST and history of previous surgery were not associated with higher Parkland Grading Scale grade. Lee et al. have compared the Parkland Grading Scale grading with preoperative factors. They reported that there was statistically significant difference in different Parkland Grading Scale grading in relation to the age, male gender, and WBC count while there was no difference in relation to BMI (p = 0.155) [29]. Similarly, Madni et al. (p = 0.0001), Abdul et al. (p = 0.0001), and Baral et al. (p = 0.0001) reported there was statistically significant difference in WBC count which was not reported in our study [26, 30, 31]. There was similar finding reported by Abdul et al. with relation to GB wall thickness and Parkland Grading Scale grading which was statistically significant [30].From these results, we can assume that the Parkland Grading Scale is as feasible as the pre-operative factors to predict the difficult LC. We believe the inflammation of the GB seen intra-operatively is more vital than the USG findings because it is simple and covers wider range of difficulty variation.

Regarding the post-operative outcomes, there was no significance in incidence of post-operative pneumonia, post-operative bile leak while the incidence of SSI, post-operative fever, and total length of post-operative stay significantly increased with Parkland Grading Scale grading. Even though we had 44(21.4%) patients with bile spillage during the surgery due to iatrogenic GB perforation, there were only 5(2.4%) patients with SSI. This may be due a thorough washing intra-operatively under clear vision. There was no SSI in the patients whose surgeries were converted to open. In our study, 3 (1.4%) patients had post-operative bile leak and there was no significant difference in co-relation to increase Parkland Grading Scale grading. Madni et al. reported 5 (1.5%) patients and Baral et al. reported 3 (1.6%) patients with post-operative bile leakage but in contrast to our study, it was statistically significant as the Parkland Grading Scale grade increased [26, 31]. Our median post-operative total stay was 2 days. Madni et al. and Abdul et al. reported significant difference in post-operative total stay as the Parkland Grading Scale grade increased which was consistent with our study [26, 31]. From all these findings, we can conclude that Parkland Grading Scale system plays important role in determining the post-operative outcomes.

There were some limitations of the study. This was a single centered study. We need to conduct multi-institutional study in larger scale to further validate the Parkland Grading Scale system.

## Conclusion

Parkland Grading Scale system is a reliable intra-operative grading system to stratify the severity of the gallbladder diseases. It is reliable to assess the difficulty in LC and helps the surgeon to change the strategy of management. Increase in Parkland Grading Scale is associated with increase severity of the GB inflammation and difficulty level of the surgery, conversion rate, length of operative time and total post-operative hospital stay.

## Data Availability

The datasets used and/or analyzed in this study are available from the corresponding author upon reasonable request.

## Abbreviations

ALP: alkaline phosphatase
ALT: Alanine transaminase
AST: Aspartate transaminase
BDI: Bile duct injury
CBD: Common bile duct
CMCTH: Chitwan Medical College Teaching Hospital
ERCP: Endoscopic retrograde cholangio-prancreaticography
GB: Gallbladder
GSD: Gallstone disease
Hb%: Hemoglobin
LC: Laparoscopic cholecystectomy
PT: Prothrombin time
SSI: Surgical site infection
WBC: White blood cell
